# Dwell-Time Distribution, Long Pausing and Arrest of Single-Ribosome Translation through the mRNA Duplex

**DOI:** 10.3390/ijms161023723

**Published:** 2015-10-09

**Authors:** Ping Xie

**Affiliations:** Key Laboratory of Soft Matter Physics and Beijing National Laboratory for Condensed Matter Physics, Institute of Physics, Chinese Academy of Sciences, Beijing 100190, China; E-Mail: pxie@aphy.iphy.ac.cn; Tel.: +86-10-8264-9387

**Keywords:** ribosome, translation, tRNA–mRNA translocation, mRNA unwinding, molecular machine

## Abstract

Proteins in the cell are synthesized by a ribosome translating the genetic information encoded on the single-stranded messenger RNA (mRNA). It has been shown that the ribosome can also translate through the duplex region of the mRNA by unwinding the duplex. Here, based on our proposed model of the ribosome translation through the mRNA duplex we study theoretically the distribution of dwell times of the ribosome translation through the mRNA duplex under the effect of a pulling force externally applied to the ends of the mRNA to unzip the duplex. We provide quantitative explanations of the available single molecule experimental data on the distribution of dwell times with both short and long durations, on rescuing of the long paused ribosomes by raising the pulling force to unzip the duplex, on translational arrests induced by the mRNA duplex and Shine-Dalgarno(SD)-like sequence in the mRNA. The functional consequences of the pauses or arrests caused by the mRNA duplex and the SD sequence are discussed and compared with those obtained from other types of pausing, such as those induced by “hungry” codons or interactions of specific sequences in the nascent chain with the ribosomal exit tunnel*.*

## 1. Introduction

The ribosome is a two-subunit macromolecular machine that synthesizes polypeptide chains, in which the sequence of the amino acid residues is dictated by that of the codons (triplets of nucleotides) on a single-stranded messenger RNA (mRNA). However, it has been shown that many mRNAs can form folded structures in their coding regions [1,2,3,4]. Thus, when translating through the duplex region of mRNA, the duplex is required to unwind for the ribosome to read the codons on the single-stranded mRNA. It has been well determined that the ribosome itself can unwind the mRNA duplex [5], without the requirement of RNA helicases, like the RNA polymerase transcription through the double-stranded DNA.

To understand the mechanism and dynamics of the ribosome translation through the mRNA duplex, besides the biochemical assays [5] different single molecule methods have been employed such as the optical trapping [6,7] and the single molecule fluorescence energy transfer (smFRET) [8,9]. Using the optical trapping method, Wen *et al.* [6] followed in real time the translation through the mRNA duplex by single ribosomes. They found that the translation occurs through successive translocation-and-pause cycles and determined the distribution of pause durations (or the distribution of dwell times). They showed that raising the pulling force applied externally to the ends of the mRNA to unzip the duplex decreases the pause durations but does not affect the translocation times; the long paused ribosomes can be rescued by raising the pulling force [6]. Moreover, the dependence of the translation rate on the pulling force was also determined [7].

In order to quantitatively explain the distribution of dwell times observed experimentally [6], Tinoco and Wen [10] and Chowdhury and colleagues [11,12] have made theoretical studies based on the model of the ribosome translation through the single-stranded mRNA. However, several relevant issues have not been studied theoretically, for example, how variations of the pulling force to unzip the mRNA duplex affect the distribution of dwell times, how pauses of long durations occur sometimes, how the presence of Shine-Dalgarno(SD)-like sequence can induce translational arrest, or how the long paused or arrested ribosomes can be rescued by raising the pulling force to unzip the mRNA duplex*.*

We recently proposed a model of the ribosome translation through the mRNA duplex, where it was proposed that the resistance arising from mRNA unwinding results in the occurrence of the futile translocation besides the usually effective translocation [13]. Based on the proposal, the theoretical results on the mean rate of the ribosome translation through the duplex region of mRNA as a function of the pulling force to unzip the duplex are in good agreement with the available experimental data [7] (see also Figure S1). In this work, based on the same proposal we study the distribution of dwell times during the ribosome translation through the mRNA duplex, addressing the above-mentioned unclear issues.

## 2. Results

### 2.1. Comparison between Theoretical and Experimental Data on the Distribution of Dwell Times

In this section, we compare the theoretical results on the distribution of dwell times with the experimental data of Wen *et al.* [6]. To fit the experimental data by using Equations (7)–(24) (see Section 4), only two parameters, *F* and *E*_bp_, are adjustable while values of other parameters $E_{\text{PE}}^{\text{(}50S\text{)}}$, *Z*_max_ and *b* (see Section 4.5) and values of rate constants *k*_1_–*k*_11_ and *k_r_* (see Section 4.5 and Table 1) are kept unchanged. Different values of *E*_bp_ correspond to different sequences (*i.e.*, different GC contents) of the mRNA duplex.

**Table 1:** *In vitro* values of rate constants defined in Figure 9 for the *Escherichia coli* ribosome.

Rate Constants	Values	Reference
*k*_1_ (s^–1^)	1	[14,15,16,17,18]
*k*_2_ (s^–1^)	0	[17,18,19]
*k*_3_ (s^–1^)	20	[20]
*k*_4_ (s^–1^)	100	[20]
*k*_5_ (s^–1^)	20	[21,22,23,24,25,26]
*k_b_* (µM^–1^∙s^–1^)	110	[21,22,23,24,25,26]
*k*_–6_ (s^–1^)	25	[21,22,23,24,25,26]
*k*_7_ (s^–1^)	100	[21,22,23,24,25,26]
*k*_–7_ (s^–1^)	0.2	[21,22,23,24,25,26]
*k*_8_ (s^–1^)	260	[21,22,23,24,25,26]
*k*_9_ (s^–1^)	60	[21,22,23,24,25,26]
*k*_10_ (s^–1^)	3	[21,22,23,24,25,26]
*k*_11_ (s^–1^)	50	[21,22,23,24,25,26]
*k_r_* (s^–1^)	1	[21,22,23,24,25,26,27]

**Figure 1:**
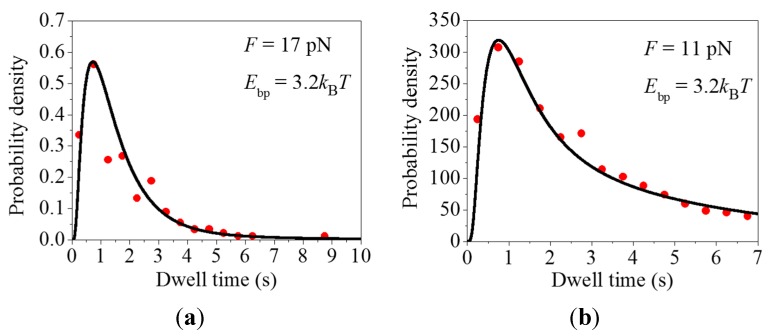
Distributions of dwell times. Lines represent the theoretical results and dots represent the experimental data taken from Wen *et al.* [6]. In order to make a direct comparison with the experimental data, we multiply the calculated distribution of dwell times, *h*(*t*), by a constant *C*. (**a**) The theoretical results are calculated with *F* = 17 pN and *E*_bp_ = 3.2*k_B_T*. The experimental data are taken from Figure 4a in Wen *et al.* [6] (reproduced with permission from Nature); (**b**) The theoretical results are calculated with *F* = 11 pN and *E*_bp_ = 3.2*k*_B_*T*. The experimental data are taken from Figure S5a in Wen *et al.* [6] (reproduced with permission from Nature).

First, we study the distribution of dwell times with short durations. Taking *F* = 17 pN and *E*_bp_ = 3.2*k*_B_*T*, the theoretical results for the distribution of dwell times are shown in Figure 1a (line), which are in good agreement with the experimental data (dots) (Figure 4a in Wen *et al.* [6] where the pulling force *F* was around 16 pN). The value of *E*_bp_ = 3.2*k*_B_*T* corresponds to the sequence of the mRNA duplex where the mRNA base pairs have an intermediate stability. Taking *F* = 11 pN and *E*_bp_ = 3.2*k*_B_*T*, the theoretical results for the distribution of dwell times are shown in Figure 1b (line), which are also in good agreement with the experimental data (dots) (Figure S5a in Wen *et al.* [6] where the pulling force *F* was smaller than 16 pN).

By comparison, we fit the experimental data with the theoretical data without consideration of the occurrence of the futile translocation, *i.e.*, taking *P*_E_ = 1 (see Section 4), as done before [10,11,12] (see Figure S2). In Figure S2a we use values of the rate constants *k*_1_–*k*_11_ as given in Table 1. In order to make the mean dwell time close to the experimental data, in Figure S2b we require dividing values of all rate constants *k*_1_–*k*_11_ given in Table 1 by 1.5. Importantly, it has been generally believed that the downstream mRNA duplex impedes the mRNA translocation while has no effect on other transitions, and thus the reduced rate of the translation through the mRNA duplex results from the reduced rate constant of the mRNA-translocation step [7]. Under this consideration, we should only decrease the rate constant of the mRNA-translocation step, *k*_4_, with values of other rate constants being unchanged, as given in Table 1. To make the mean dwell time close to the experimental data, we requires dividing *k*_4_ by 80 in Figure S2c.

Comparing Figure 1 with Figure S2, we see that the experimental data (especially the data in Figure S5a of Wen *et al.* [6]) are fitted worse with model of *P*_E_ = 1 (Figure S2) than with our model of $P_{\text{E}}^{\left(n\right)}$ < 1 (here *n* < 3) (Figure 1). In particular, the theoretical data in Figure S2c deviates significantly from the experimental data. Quantitatively, the mean relative difference between the experimental and the theoretical data Δ ≈ 0.25 for Figure 1a, whereas Δ ≈ 0.44 for Figure S2a; Δ ≈ 0.10 for Figure 1b, whereas Δ ≈ 0.36 for Figure S2b and Δ ≈ 0.44 for Figure S2c. Here, the mean relative difference is calculated by $\Delta =\sum_{i=1}^{N}[(|h_{i}^{\left(\exp\right)}-h_{i}^{\left(\mathrm{theo}\right)}|)/h_{i}^{\left(\exp\right)}]/N$, where $h_{i}^{\left(\exp\right)}$ and $h_{i}^{\left(theo\right)}$ are the experimental and theoretical data respectively for the distribution at point *i* of the total *N* = 14 points shown in the figures. In addition, it is noted that it is unreasonable that the downstream mRNA duplex can cause all of the rate constants *k*_1_–*k*_11_ in Figure S2b to become smaller than those in Figure S2a where no downstream mRNA duplex is present.

**Figure 2:**
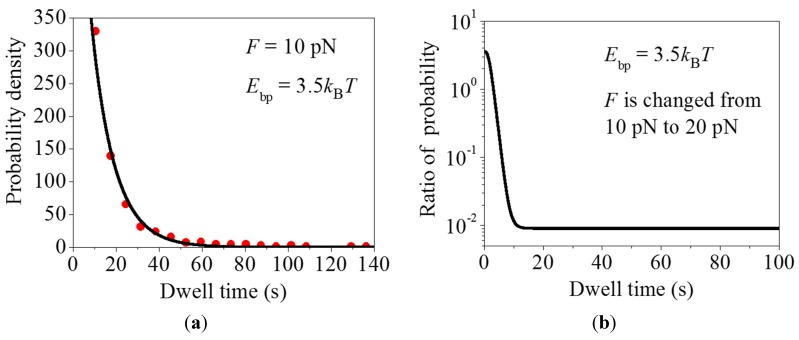
Dynamics of long pauses. (**a**) Distribution of dwell times with long durations (>7 s). Line represents the theoretical results calculated with *F* = 10 pN and *E*_bp_ = 3.5*k_B_T* and dots are experimental data taken from Figure S5b in Wen *et al.* [6] (reproduced with permission from Nature). In order to make a direct comparison with the experimental data, we multiply the calculated distribution of dwell times, *h*(*t*), by a constant *C*; and (**b**) Ratio of the occurrence probability of the dwell time calculated with *F* = 20 pN to that calculated with *F* = 10 pN, where *E*_bp_ = 3.5*k*_B_*T*. The ratio is obtained by dividing *h*(*t*) that is calculated by using Equation (24) with *F* = 20 pN by *h*(*t*) that is calculated with *F* = 10 pN.

Second, we study the distribution of dwell times with long durations (>7 s). Taking *F* = 10 pN and *E*_bp_ = 3.5*k*_B_*T*, the theoretical results for the distribution of dwell times with long durations are shown in Figure 2a (line), which are also in agreement with the experimental data (dots) (Figure S5b in Wen *et al.* [6]). The value of *E*_bp_ = 3.5*k*_B_*T* corresponds to the sequence of the mRNA duplex where the mRNA base pairs have a relatively strong stability. If the pulling force *F* = 10 pN is increased to a larger value of 20 pN, the occurrence probability of the dwell time with a long duration (e.g., ≥20 s) is reduced by more than 100-fold, as seen from Figure 2b, where we show the ratio of the occurrence probability of the dwell time calculated with *F* = 20 pN to that calculated with *F* = 10 pN. These results imply that the long paused ribosomes can be rescued by raising the pulling force *F*, which is consistent with the experimental data [6]. In addition, in our model whether under the low pulling force or under the large pulling force, the rate constant of the translocation step, *k*_4_, always has the large value, which is also consistent with single molecule experimental data showing that raising the pulling force decreases the pause durations but does not affect the translocation times [6].

By comparison, in order to explain the experimental data on the distribution of dwell times with long durations (>7 s) without consideration of the occurrence of the futile translocation, *i.e.*, taking *P*_E_ = 1, we require dividing values of *k*_1_–*k*_11_ given in Table 1 by 9 (see Figure S3a) or dividing the value of *k*_4_ by 1000 while taking values of other rate constants as given in Table 1 (see Figure S3b). However, it is unreasonable that the downstream mRNA duplex can cause all of the rate constants *k*_1_–*k*_11_ in Figure S3a to become smaller than those in Figure S2a where there is no downstream mRNA duplex. Although by reducing *k*_4_ greatly (by 1000-fold) the theoretical data for the distribution of dwell times with long durations can be consistent with the experimental data (Figure S3b), the theoretical data for the distribution of dwell times with short durations deviate significantly from the experimental data (Figure S2c). Moreover, reducing *k*_4_ by 1000-fold gives the rate constant of the translocation step to be 0.1 s^–1^, which is much smaller than that (100 s^–1^) when a large pulling force is applied to the mRNA to unzip the duplex. This is inconsistent with single molecule experimental data showing that raising the pulling force decreases the pause durations but does not affect the translocation times [6].

### 2.2. Effect of the Pulling Force to Unzip the mRNA Duplex on the Distribution of Dwell Times

**Figure 3:**
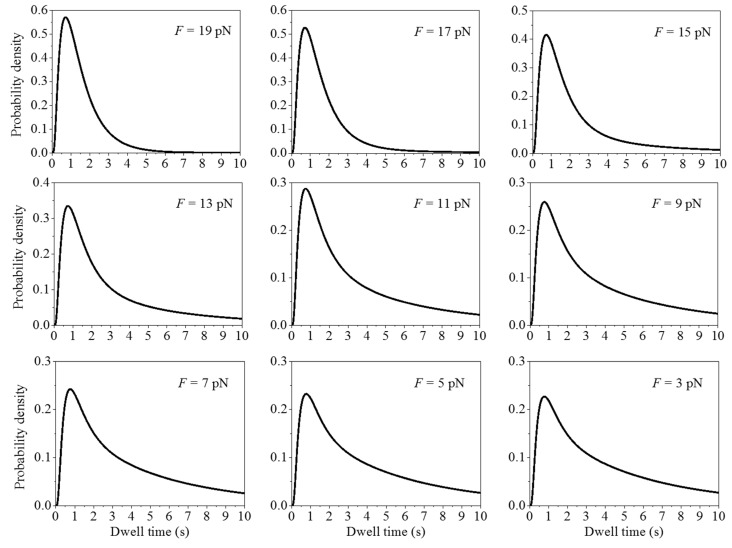
Normalized distributions of dwell times, *h*(*t*), calculated with different values of *F*, where *E*_bp_ = 3.2*k*_B_*T*.

**Figure 4:**
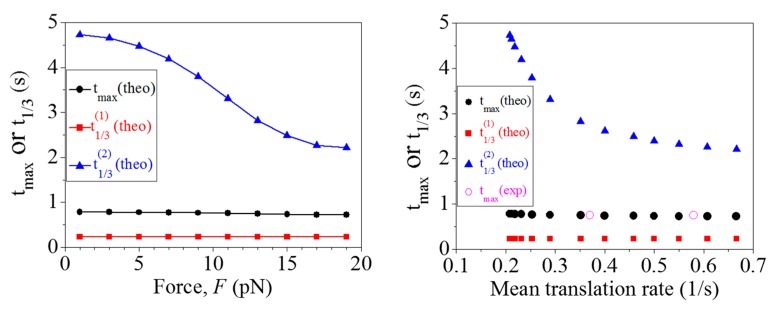
Dwell time, *t*_max_, at which the maximum value of the distribution occurs, dwell time, $t_{1/3}^{\left(1\right)}$, at which the distribution increases from zero to the value equal to one third of the maximal value, and dwell time, $t_{1/3}^{\left(2\right)}$, at which the distribution decreases from the maximal value to the value equal to one third of the maximal value, *vs.* *F* (**left** panel), which are obtained from Figure 5. The **right** panel corresponds to *t*_max_, $t_{1/3}^{\left(1\right)}$ and $t_{1/3}^{\left(2\right)}$ *vs.* the mean translation rate *v*, where the theoretical data for *v* are calculated by using Equations (S1), (S4) and (S7). The two experimental datasets for *t*_max_ are taken from and those for *v* are calculated from the two distributions of dwell times shown in Figure 4a and Figure S5a of Wen *et al.* [6].

In this section, we calculate systematically the distributions of dwell times under different values of the pulling force *F* to unzip the mRNA duplex with fixed *E*_bp_ = 3.2*k*_B_*T*. Some results are shown in Figure 3. It is seen that at *t* ≤ *t*_max_, the curve form for the distribution is insensitive to *F*, where *t*_max_ corresponds to the dwell time at which the maximum value of the distribution occurs. This can also be seen from Figure 4, showing that *t*_max_ and $t_{1/3}^{\left(1\right)}$ are insensitive to *F* (Figure 4a) or *t*_max_ and $t_{1/3}^{\left(1\right)}$ are insensitive to the mean translation rate (Figure 4b), where $t_{1/3}^{\left(1\right)}$ corresponds to the dwell time at which the distribution increases from zero to the value equal to one third of the maximal value. By contrast, at *t* > *t*_max_, the distribution becomes wider as *F* becomes smaller (Figure 3). This can also be seen from Figure 4, showing that $t_{1/3}^{\left(2\right)}$ increases with the decrease of *F* (Figure 4a) or $t_{1/3}^{\left(2\right)}$ increases with the decrease of the mean translation rate (Figure 4b), where $t_{1/3}^{\left(2\right)}$ corresponds to the dwell time at which the distribution decreases from the maximal value to the value equal to one third of the maximal value.From Figure 4b, it is seen that the two available experimental data for *t*_max_ are in good agreement with the theoretical data. The other predicted results shown in Figure 4a,b can be easily tested by future experiments. The interesting characteristic of our results (Figure 4) is that *t*_max_ is nearly independent of *F*. This is because the dwell time, $t_{\max}^{\left(n\right)}$, at which the maximum value of the distribution *h*^(n)^(*t*) occurs, is nearly independent of $P_{\text{E}}^{\text{(}n\text{)}}$ (see Figures S6 and S7), giving the overall distribution of dwell times *h*(*t*) (see Equation (23)) having the value of *t*_max_ being nearly independent of *F*.

**Figure 5:**
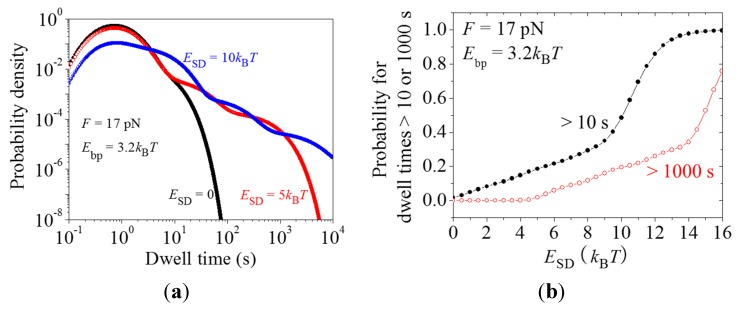
Dynamics of translation through the mRNA duplex in the presence of the interaction of the SD sequence with the anti-SD site of the 30S subunit. (**a**) Normalized distributions of dwell times, *h*(*t*), calculated with different values of the binding energy between SD and anti-SD, *E*_SD_, where *F* = 17 pN and *E*_bp_ = 3.2*k*_B_*T*; and (**b**) Probability of pausing (>10 s) and probability of arrest (>1000 s) *vs. E*_SD_.

By comparison, it is interesting to see the results for the distributions of dwell times calculated with *P*_E_ = 1 but by decreasing the rate constant of the mRNA-translocation step, *k*_4_, by different times and with values of other rate constants being unchanged. This corresponds to the case with the assumption that the downstream mRNA duplex impedes the mRNA movement while has no effect on other transitions and thus the reduced rate of the translation through the mRNA duplex results from the reduced rate constant of the mRNA-translocation step [7], as mentioned above. As shown in Qu *et al.* [7], the mean rate of translation through the mRNA duplex depends on the pulling force *F* to unzip the duplex and on the stability of the duplex (*i.e.*, the GC content of the duplex). Under the above assumption, this implies that the change in the value of *k*_4_ can be achieved experimentally by changing *F* and/or the GC content of the duplex. In Figure S4 we show some results for the distributions of dwell times by decreasing *k*_4_ by different times α (*i.e.*, with *k*_4_ being replaced by *k*_4_/α). From Figure S4, it is seen that changing *k*_4_ affects sensitively the distribution at both *t* ≤ *t*_max_ and *t* > *t_max_*, with $t_{1/3}^{\left(1\right)}$, *t*_max_ and $t_{1/3}^{\left(2\right)}$ increasing sensitively with the increase of α or $t_{1/3}^{\left(1\right)}$, *t*_max_ and $t_{1/3}^{\left(2\right)}$ decreasing sensitively with the increase of the translation rate (see Figure S5). This effect of changing *k*_4_ on the distribution of dwell times (Figures S4 and S5) is in sharp contrast to that of changing $P_{\text{E}}^{\left(n\right)}$ as shown in Figures S6 and S7 or to that of changing the overall probability of effective translocation via changing *F* as shown in Figure 3 and Figure 4. In addition, from Figure S5b it is seen that the two available experimental data for *t*_max_ deviate significantly from the theoretical data.

### 2.3. Dynamics of Pausing and Arrest Caused by the Interaction of the SD Sequence with Anti-SD Site of the 30S Subunit

In this section, we study the effect of the SD sequence in the mRNA on the distribution of dwell times, pausing and arrest during the translation through the mRNA duplex and/or the single-stranded mRNA. First, consider the translation through the mRNA duplex. In the absence of the interaction between the upstream SD-like sequence and the anti-SD site of the 30S subunit, the probability of the effective translocation is calculated by Equations (7) and (8). In the presence of the interaction, Equations (7) and (8) are replaced by:

(1) $$P_{\text{E}}^{\left(n\leq 2\right)}=\frac{\exp\left\{-\left[(3-n)E_{\text{bp}}+E_{\text{SD}}\right]/k_{\text{B}}T\right\}}{\exp\left\{-\left[(3-n)E_{\text{bp}}+E_{\text{SD}}\right]/k_{\text{B}}T\right\}+\exp\left(-E_{\text{PE}}^{\left(50S\right)}/k_{\text{B}}T\right)}$$

(2) $$P_{\text{E}}^{\left(n\geq 3\right)}=\frac{\exp\left(-E_{\text{SD}}/k_{\text{B}}T\right)}{\exp\left(-E_{\text{SD}}/k_{\text{B}}T\right)+\exp\left(-E_{\text{PE}}^{\left(50S\right)}/k_{\text{B}}T\right)}$$

where *E*_SD_ represents the binding energy between SD and anti-SD, while the other Equations (9)–(24) presented above are kept unchanged.

Taking *F* = 17 pN and *E*_bp_ = 3.2*k*_B_*T*, with Equations (1), (2), and (9)–(24) the calculated results for the distribution of dwell times for different values of *E*_SD_ are shown in Figure 5a. It is seen that the presence of *E*_SD_ significantly widens the distribution at *t* > *t*_max_. As a result, the probability of the dwell times with long durations (≥10 s) can be increased greatly in the presence of SD (Figure 5b). Moreover, the translation arrest, which is defined here as the dwell times longer than 1000 s, can also occur frequently when *E*_SD_ has large values (Figure 5b). In Figure 5b, the probability of pausing or arrest is calculated by $\int_{T_{0}}^{\infty }h\left(t\right)dt$ or by $1-\int_{0}^{T_{0}}h\left(t\right)dt$, where *T*_0_ = 10 s and 1000 s for pausing and arrest, respectively. These results (Figure 5b) are consistent with the single molecule experimental data of Wen *et al.* [6].

Then, consider the translation through the single-stranded mRNA. In the presence of *E_SD_*, Equations (7) and (8) are replaced by

(3) $$P_{\text{E}}=\frac{\exp\left(-E_{\text{SD}}/k_{\text{B}}T\right)}{\exp\left(-E_{\text{SD}}/k_{\text{B}}T\right)+\exp\left(-E_{\text{PE}}^{\left(50S\right)}/k_{\text{B}}T\right)}$$

**Figure 6:**
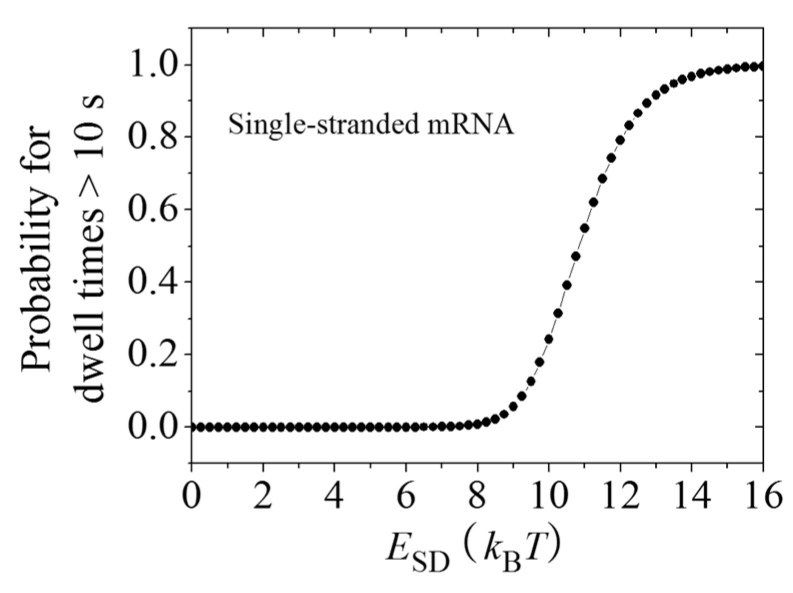
Dynamics of translation through the single-stranded mRNA in the presence of the interaction of the SD sequence with the anti-SD site of the 30S subunit. Probability of pausing (>10 s) *vs.* the binding energy between the SD and anti-SD, *E*_SD_.

With Equations (3) and (9)–(24), the calculated results for the probability of pausing (>10 s) *vs. E*_SD_ are shown in Figure 6. When *E*_SD_ is smaller than $E_{\text{PE}}^{\left(50S\right)}$ = 9*k*_B_*T*, the pausing rarely occurs. However, when *E*_SD_ becomes larger than $E_{\text{PE}}^{\left(50S\right)}$, the occurrence probability of the pausing increases significantly with the increase of *E*_SD_. This provides an explanation of the recent experimental data of Li *et al.* [28] showing that the presence of SD sequence in the mRNA would also induce pausing of the ribosome when translating through the single-stranded mRNA.

**Figure 7:**
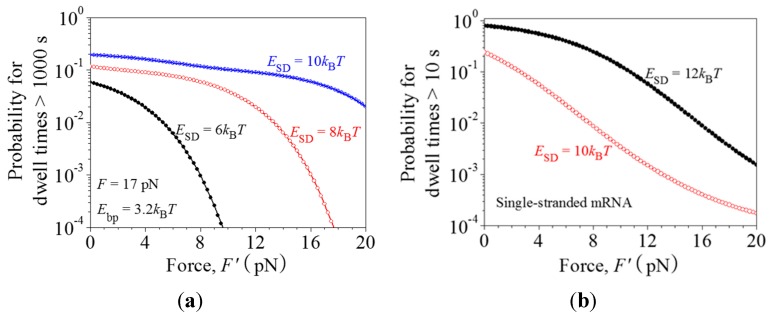
Ribosomal arrest or pausing caused by the interaction of the SD sequence with the anti-SD site of the 30S subunit can be rescued by external force *F*′ applied to the 30S subunit. (**a**) Probability of arrest (>1000 s) *vs.* force *F*′ for the ribosome translation through the mRNA duplex with different values of the binding energy between SD and anti-SD, *E*_SD_. *F* = 17 pN and *E*_bp_ = 3.2*k*_B_*T*; (**b**) Probability of pausing (>10 s) *vs.* force *F*′ for the ribosome translation through the single-stranded mRNA with different values of *E_SD_*.

As shown above (see Figure 2b), raising the pulling force *F* to unzip the mRNA duplex can rescue the paused ribosome. Similarly, we consider an external force, *F*′, acting on the body of the 30S subunit of the translating ribosome to rescue the paused ribosome. The external force *F*′ can be realized by using an optical single-trapping assay in which the body of the 30S subunit is fixed to a solid surface and the end of the upstream mRNA is attached to a micrometre-sized bead held in the optical trap. For translation through the mRNA duplex, under the force *F*′ Equations (1) and (2) are replaced by

(4) $$P_{\text{E}}^{\left(n\leq 2\right)}=\frac{\exp\left\{-\left[(3-n)E_{\text{bp}}+E_{\text{SD}}-F\prime d\right]/k_{\text{B}}T\right\}}{\exp\left\{-\left[(3-n)E_{\text{bp}}+E_{\text{SD}}-F\prime d\right]/k_{\text{B}}T\right\}+\exp\left(-E_{\text{PE}}^{\left(50S\right)}/k_{\text{B}}T\right)}$$

(5) $$P_{\text{E}}^{\left(n\geq 3\right)}=\frac{\exp\left[-\left(E_{\text{SD}}-F\prime d\right)/k_{\text{B}}T\right]}{\exp\left[-\left(E_{\text{SD}}-F\prime d\right)/k_{\text{B}}T\right]+\exp\left(-E_{\text{PE}}^{\left(50S\right)}/k_{\text{B}}T\right)}$$

where *d* = 3*p*, with *p* = 0.34 nm being the distance between two successive nucleotides on the single-stranded mRNA. With Equations (4), (5) and (9)–(24), the calculated results for the probability of translation arrest (>1000 s) *vs.* force *F*′ for different values of *E*_SD_ are shown in Figure 7a, where we take *F* = 17 pN and *E*_bp_ = 3.2*k*_B_*T*. It is clear that a large force *F*′ can significantly reduce the probability of translation arrest.

Similarly, for translation through the single-stranded mRNA, under the force *F*′ Equation (3) is replaced by

(6) $$P_{\text{E}}=\frac{\exp\left[-\left(E_{\text{SD}}-F\prime d\right)/k_{\text{B}}T\right]}{\exp\left[-\left(E_{\text{SD}}-F\prime d\right)/k_{\text{B}}T\right]+\exp\left(-E_{\text{PE}}^{\left(50S\right)}/k_{\text{B}}T\right)}$$

With Equations (6) and (9)–(24), the calculated results for the probability of pausing (>10 s) *vs.* force *F*′ for different values of *E*_SD_ are shown in Figure 7b. It is also seen that a large force *F*′ can significantly reduce the probability of pausing.

## 3. Discussion

### 3.1. The Justification of the Occurrence of Futile Translocation and Multiple GTP Hydrolyses

**Figure 8:**
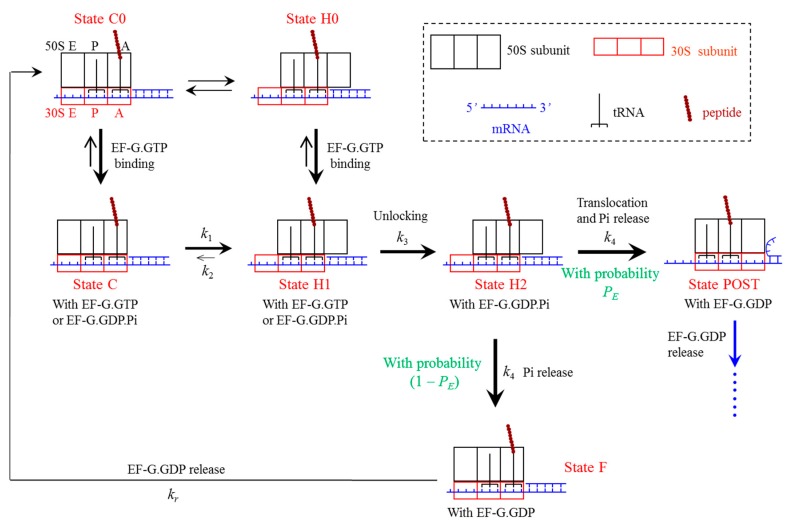
Model of ribosomal translocation through the mRNA duplex (see text for detailed description).

**Figure 9:**
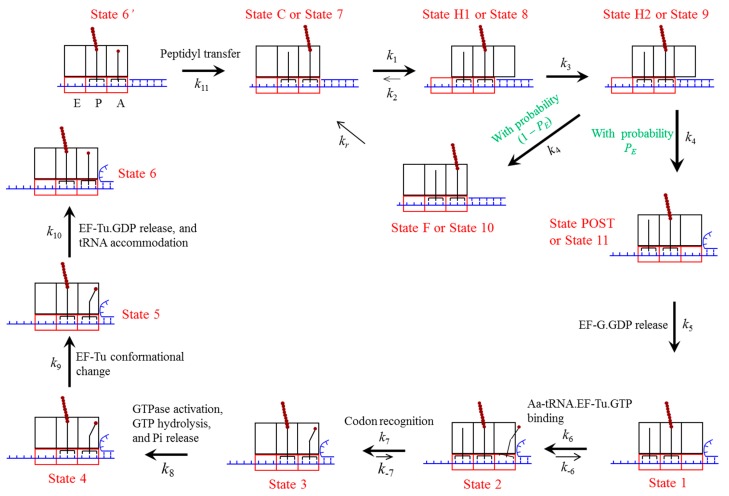
Pathway of the ribosome translation elongation through the mRNA duplex at saturating concentration of EF-G∙GTP (see text for detailed description). The rate constant *k*_6_ = *k*_b_ [TC], where *k*_b_ is the binding rate of the aminoacyl-tRNA∙EF-Tu∙GTP ternary complex and [TC] is the concentration of the ternary complex. Here, we drew deacylated tRNA dissociation after the binding of the ternary complex although the dissociation can occur at any state after the post-translocation.

It has been shown that the single molecule optical trapping data [7] on the rate of ribosome translation through the duplex region of mRNA as a function of the pulling force to unzip the duplex can be quantitatively explained by using the translation model shown in Figure 8 and Figure 9 [13], where besides the effective translocation the futile translocation can also occur due to the resistance resulting from mRNA unwinding. Moreover, with the occurrence of futile translocation the smFRET data on the effect of the downstream mRNA secondary structure on deacylated tRNA dissociation [8] and in particular, on multiple fluctuations of the ribosomal complex between classical non-rotated and rotated/hybrid states before undergoing mRNA translocation through the mRNA duplex at saturating EF-G∙GTP [9] can also be explained quantitatively [29,30]. In the current work, we show that with the occurrence of futile translocation, the single molecule optical trapping data on the distribution of dwell times [6] can also be explained well (Figure 1, Figure 2 and Figure 4).

The occurrence of futile translocation can also be justified from the following considerations. Suppose that no transition of State H2 to State F occurs in Figure 8. Due to the obstacle arising from the downstream mRNA duplex, the ribosomal complex would pause at State H2, with the mRNA movement being delayed for a long time. The available biochemical data with the single-stranded mRNA showed that after ribosomal unlocking, Pi release takes place rapidly and independently of blocking the mRNA movement [20]. It would be then expected that for the case of translocation through the mRNA duplex, during the long pausing period of mRNA movement Pi still releases rapidly and thus State H2 bound with EF-G∙GDP∙Pi changes rapidly to the state bound with EF-G∙GDP long before the mRNA movement takes place. On the other hand, the available experimental data showed convincingly that in the presence of EF-G∙GDP, no mRNA movement can occur [31] (see also detailed discussion in reference [29]). Thus, after the long pause the mRNA movement cannot occur in the presence of EF-G∙GDP and only after EF-G∙GDP release and then EF-G∙GTP rebinding can the mRNA movement takes place, implying that multiple GTP hydrolyses are also required. However, if this supposition is true, the ribosomal complex would sample the hybrid state only once before undergoing mRNA translocation at saturating EF-G∙GTP, which is inconsistent with the smFRET data of Kim *et al.* [9]. In addition, as shown in the current work, if the supposition is true, the theoretical results for the distribution of dwell times deviate significantly from the single molecule optical trapping data (Figures S2 and S5). By contrast, the occurrence of futile translocation can give quantitative explanations of the smFRET data on multiple fluctuations between non-rotated conformation (State C and State F, Figure 9) and rotated conformation (State H1 and State H2, Figure 9) before undergoing mRNA translocation at saturating EF-G∙GTP [30]. Moreover, the single molecule optical trapping data on the distribution of dwell times can also be fitted well (Figure 1, Figure 2 and Figure 4). These comparisons between the theoretical analyses and the experimental data argue against the assumption that the long pausing arises from the reduced rate of mRNA movement but support the proposal of the existence of State F.

### 3.2. Three Types of Translational Pausing

We quantitatively explain the dynamics of long pauses during the ribosome translation through the mRNA duplex and explain the translational pausing caused by the SD-like sequence at saturating ternary complex. These pauses caused by the mRNA duplex and/or the SD-like sequence are called type-I pausing here. It is interesting to note that in the long period of type-I pausing, EF-G∙GTP binding, GTP hydrolysis and EF-G∙GDP release still occur and the GTP hydrolysis induces the futile translocations. This is in contrast to the transcription elongation by RNA polymerases, where in the long pausing period the chemical reaction of the nucleotide incorporation does not occur [32]. However, for both the ribosome translation through the mRNA duplex and the RNA polymerase transcription elongation, these long pauses can be rescued by a large force that facilitates the forward translocation. In addition, it has been suggested that in living cells the difference in the concentration of the cognate ternary complex could cause different translation rates at different codons, resulting in the translational pausing at the “hungry” codon with extremely low concentration of the cognate ternary complex [33,34], which is called type-II pausing here. It is noted that in the period of type-II pausing, the chemical reactions of EF-G∙GTP binding, GTP hydrolysis, EF-G∙GDP release as well as tRNA decoding and then peptidyl transfer do not occur, with the characteristic being similar to the transcription elongation by RNA polymerases. Moreover, it is noted that the long type-II translational pauses cannot be rescued by a large force that facilitates the forward process, which is contrast to type-I pauses studied in the current work and the pauses of transcription elongation by RNA polymerases. Another difference between type-I and type-II translational pausing is that the pauses take place at different steps in an elongation cycle, with the former taking place at the mRNA translocation step and the latter taking place at the step of cognate ternary-complex binding. Besides type-I and type-II translational pausing, it has been revealed that interactions of specific nascent chain sequences with the ribosome exit tunnel can also cause pausing [35,36], which is called type-III pausing here. This type-III pausing is caused by the repression of the peptidyl transfer, *i.e.*, the pause takes place at the step of the peptidyl transfer. Thus, it is noted that type-I, type-II and type-III pauses take place respectively at steps of translocation, tRNA binding and peptidyl transfer, which are the three typical steps in the elongation cycle. In the period of type-III pausing, the chemical reactions of EF-G∙GTP binding, GTP hydrolysis, EF-G∙GDP release as well as tRNA decoding do not occur, which is similar to type-II pausing but is in contrast to type-I pausing.

## 4. Methods

### 4.1. Model of Ribosome Translocation through the mRNA Duplex

The simplified model of ribosome translocation through the mRNA duplex catalyzed by elongation factor G (EF-G) hydrolyzing guanosine triphosphate (GTP) is presented in Figure 8 [13], which is modified from that of the translocation through the single-stranded mRNA [37]. Before EF-G∙GTP binding to the pretranslocation ribosomal complex with deacylated tRNA bound to the P (peptidyl) site of the small 30S ribosomal subunit and the peptidyl-tRNA bound to the 30S A (aminoacyl) site, the 30S subunit can spontaneously rotate counterclockwise (viewed from the exterior of the 30S) relative to the large 50S subunit and *vice verse* [14,15,16,17,18,19], which are called forward and reverse intersubunit rotations, respectively. The spontaneous intersubunit rotations induce the ribosomal complex to transit between the classical non-rotated state (State C0) and hybrid state (State H0), with the two states being in thermodynamic equilibrium with each other [14,15,16,17,18]. EF-G∙GTP can bind to both State C0 and State H0 [27,38,39].

First, consider EF-G∙GTP binding to State H0, becoming State H1. After rapid GTP hydrolysis to guanosine diphosphate and inorganic phosphate (GDP∙Pi) smaller conformational changes in tip of domain IV of EF-G bring about the forward 30S head rotation relative to the 30S body, opening the mRNA channel (termed ribosomal unlocking) (State H2) [40,41]. The ribosomal unlocking facilitates the reverse intersubunit rotation. If no downstream duplex is present, *i.e.*, no resistance is present to impede the downstream movement of the 30S subunit along the mRNA, the reverse intersubunit rotation would cause the 30S subunit to move downstream relative to the mRNA that is coupled with the two tRNAs by one codon, while the high affinity of the 50S E and P sites for the deacylated tRNA [42] and peptidyl-tRNA [43], respectively, fixes the two tRNAs to the 50S subunit. This leads to the transition of the hybrid state (State H2) to the post-translocation state (State POST). However, if the downstream duplex with an extremely strong stability or an obstacle bound very strongly to the mRNA is present, the duplex or obstacle would prevent the 30S subunit from moving downstream relative to the mRNA that is coupled with the two tRNAs. Thus, the reverse intersubunit rotation would have to cause the 50S subunit to move relative to the two tRNAs by overcoming the finite total affinity of the 50S E and P sites for the two tRNAs [42,43]. This leads to the transition of the hybrid state (State H2) to a classical non-rotated pre-translocation state (called the futile state, which is denoted by State F). Consequently, for the real case with the downstream duplex of an intermediate stability, from State H2 either the transition to State POST by unwinding three mRNA base pairs (with a probability *P*_E_) or the transition to State F by overcoming the free energy of the two tRNAs binding to the 50S E and P sites (with a probability 1–*P*_E_) can be caused by the same reverse intersubunit rotation (with rate constant *k*_4_). Here, the transition to State F is called futile translocation while the transition to State POST is called effective translocation. Facilitated by the ribosomal unlocking, Pi is also released rapidly and independently of the reverse intersubunit rotation [20]. Second, consider EF-G∙GTP binding to State C0, becoming State C. The binding of EF-G facilitates mildly the transition of State C to State H1 [18] and the hybrid State H1 is then stabilized [14,17,18,19]. In State H1 the ribosomal unlocking occurs (becoming State H2). From State H2 either the transition to State POST or the transition to State F occurs, as discussed above. After transition to the non-rotated conformation (either State POST or State F), the mRNA channel in the 30S subunit becomes tight again, as proposed before [41,44]. If transition to State F occurs, after EF-G∙GDP is released, State F becomes State C0. Then, the next round of transitions from State C0 through State H2 proceeds.

The peculiarity of the translocation model is the existence of State F (the futile state), where the ribosomal complex is in the non-rotated pretranslocation conformation, with the deacylated tRNA and peptidyl-tRNA being in the P site of both subunits (P/P site) and A/A site, respectively. This is in contrast to the classical non-rotated posttranslocation conformation (State POST), where the deacylated tRNA and peptidyl-tRNA are in the E/E and P/P sites, respectively. State F has a similar structure to the classical non-rotated pretranslocation state (State C). The difference between them is that State F is bound with EF-G∙GDP while State C is bound with EF-G∙GTP or EF-G∙GDP∙Pi. The justification of the existence of State F during translocation through the mRNA duplex is discussed in Discussion (see Section 3.1).

### 4.2. Elongation Cycle of Ribosome Translation through the mRNA Duplex at Saturating Concentration of EF-G∙GTP

Based on the translocation model (Figure 8), the elongation pathway of translation through the mRNA duplex at saturating concentration of EF-G∙GTP is shown in Figure 9. Consider just after the peptidyl transfer in an elongation cycle. The ribosomal complex is in the classical non-rotated pretranslocation state (State C0, which is not shown in Figure 9 because it is short-lived at saturating EF-G∙GTP). Then, EF-G∙GTP of saturating concentration binds immediately to the classical non-rotated pretranslocation state (becoming State C) before State C0 transits spontaneously to hybrid state (State H0, which is not shown in Figure 9 because it rarely occurs in the elongation cycle under saturating EF-G∙GTP). State C then transits to State H1, where the ribosomal unlocking occurs, opening the mRNA channel (State H2). The subsequent reverse intersubunit rotation causes either effective translocation (State POST) or futile translocation (State F). If transition from State H2 to State F occurs, after the release of EF-G∙GDP, EF-G∙GTP of saturating concentration binds immediately to the pretranslocation state (becoming State C). Then, the ensuing transitions proceeds as just discussed above.

If transition from State H2 to State POST occurs, after the release of EF-G∙GDP (State 1), the ternary complex consisting of the aminoacyl-tRNA, elongation factor Tu (EF-Tu) and GTP binds to the ribosome in the partially bound A/T state (State 2). Then, the codon recognition (State 3) triggers GTPase activation, GTP hydrolysis and Pi release (State 4) [45], which is followed by a large conformational change of EF-Tu (State 5) [46,47]. EF-Tu∙GDP is then released and the aminoacyl-tRNA is accommodated into the full A/A state (State 6 or State 6′), where State 6′ is the same as State 6 except that the peptidyl-tRNA in the P/P site is prolonged by one amino acid and the mRNA is moved downstream by one codon. Now, the aminoacyl-tRNA in the A/A site reacts with the peptidyl-tRNA in the P/P site to form a peptide bond, resulting in deacylated tRNA in the P/P site and the peptidyl-tRNA prolonged by one amino acid in the A/A site (State C0). Then, the next elongation cycle proceeds.

### 4.3. Equations for Probability of Effective Translocation during Ribosome Translation through the Duplex Region of mRNA

As done in the single molecule optical trapping experiments [6,7], we consider a pulling force, *F*, applied to the ends of the mRNA duplex to unzip the duplex. If *n* ≤ 2 (*n* = 0, 1 and 2) mRNA base pairs next to the mRNA-entry channel of the ribosome are open spontaneously, which is induced by the thermal noise and the pulling force *F*, the downstream translocation of the ribosome by one codon (*i.e.*, three nucleotides) requires unwinding (3–*n*) mRNA base pairs. Then, the occurrence probability of the effective translocation, *i.e.*, the transition from State H2 to State POST (Figure 8 and Figure 9), can be calculated by

(7) $$P_{\text{E}}^{\left(n\leq 2\right)}=\frac{\exp\left[-(3-n)E_{\text{bp}}/k_{\text{B}}T\right]}{\exp\left[-(3-n)E_{\text{bp}}/k_{\text{B}}T\right]+\exp\left(-E_{\text{PE}}^{\left(50S\right)}/k_{\text{B}}T\right)}$$

where *E*_bp_ is the free energy change of unwinding an mRNA base pair, *k*_B_*T* is the thermal energy, and $E_{\text{PE}}^{\text{(50S)}}$ is the difference between the high binding energy of the 50S E and P sites for the two tRNAs and the low binding energy of the 30S subunit with the open mRNA channel for the mRNA-tRNA complex. If *n* ≥ 3 mRNA base pairs next to the mRNA-entry channel are open spontaneously, no mRNA base pair is required to unwind during the downstream translocation of the ribosome by one codon. Then, the occurrence probability of the effective translocation can be calculated by

(8) $$P_{\text{E}}^{\left(n\geq 3\right)}=\frac{1}{1+\exp\left(-E_{\text{PE}}^{\left(50S\right)}/k_{\text{B}}T\right)}$$

Under the pulling force *F*, the probability for the mRNA duplex to be open *n* (*n* = 0, 1, ⋅⋅⋅, ∞) base pairs spontaneously can be calculated by [48]

(9) $$f_{\text{O}}^{\left(n\right)}=\frac{\exp\left[-\left(nE_{\text{bp}}-E_{\text{F}}^{\left(n\right)}\right)/k_{\text{B}}T\right]}{\sum_{j=0}^{\infty }\exp\left[-\left(jE_{\text{bp}}-E_{\text{F}}^{\left(j\right)}\right)/k_{\text{B}}T\right]}$$

(10) $$E_{F}^{\left(n\right)}=2nk_{\text{B}}T\frac{Z_{\max}}{b}\ln\left[\frac{k_{\text{B}}T}{Fb}\sinh\left(\frac{Fb}{k_{\text{B}}T}\right)\right]$$

where *Z*_max_ is the maximum extension of the single-stranded mRNA containing one nucleotide under the pulling force *F* and *b* is Kuhn length of the single-stranded mRNA.

### 4.4. Equations for Distribution of Dwell Times

Based on the elongation pathway (Figure 9), for an mRNA-duplex conformation with *n* base pairs next to the mRNA-entry channel of the ribosome being open spontaneously, the distribution of dwell times, *h*^(*n*)^(*t*), at saturating concentrations of EF-G∙GTP and the ternary complex can be calculated by using the following equations

(11) $$\frac{dP\prime_{6}(t)}{dt}=-k_{11}P_{6}(t)$$

(12) $$\frac{dP_{7}(t)}{dt}=k_{11}P\prime_{6}(t)-k_{1}P_{7}(t)+k_{2}P_{8}(t)+k_{r}P_{10}(t)$$

(13) $$\frac{dP_{8}(t)}{dt}=k_{1}P_{7}(t)-\left(k_{2}+k_{3}\right)P_{8}(t)$$

(14) $$\frac{dP_{9}(t)}{dt}=k_{3}P_{8}(t)-P_{\text{E}}^{\left(n\right)}k_{4}P_{9}(t)-\left(1-P_{\text{E}}^{\left(n\right)}\right)k_{4}P_{9}(t)$$

(15) $$\frac{dP_{10}(t)}{dt}=\left(1-P_{\text{E}}^{\left(n\right)}\right)k_{4}P_{9}(t)-k_{r}P_{10}(t)$$

(16) $$\frac{dP_{11}(t)}{dt}=P_{\text{E}}^{\text{(}n)}k_{4}P_{9}(t)-k_{5}P_{11}(t)$$

(17) $$\frac{dP_{2}(t)}{dt}=k_{5}P_{11}(t)-k_{7}P_{2}(t)+k_{-7}P_{3}(t)$$

(18) $$\frac{dP_{3}(t)}{dt}=k_{7}P_{2}(t)-\left(k_{-7}+k_{8}\right)P_{3}(t)$$

(29) $$\frac{dP_{4}(t)}{dt}=k_{8}P_{3}(t)-k_{9}P_{4}(t)$$

(20) $$\frac{dP_{5}(t)}{dt}=k_{9}P_{4}(t)-k_{10}P_{5}(t)$$

(21) $$\frac{dP_{6}(t)}{dt}=k_{10}P_{5}(t)$$

where we consider that an elongation cycle begins at State 6′, with *P*′_6_ denoting the probability of State 6′ at the beginning of the elongation cycle, *P*_6_ denoting the probability of State 6 at the end of the elongation cycle and *P_i_* (*i* = 7, 8, 9, 10, 11, 1, 2, 3, 4, 5) denoting the probability of State *i* defined in Figure 9. Note that since at saturating concentration of the ternary complex the lifetime of State 1 (Figure 9) is much shorter than other states, the equation for *P*_1_(*t*) is not required to consider here for the translation at saturating ternary complex. The initial conditions at *t* = 0 are imposed as follows: *P*′_6_(0) = 1, *P_i_*(0) = 0 and *P*_6_(0) = 0.The probability density (or distribution) of dwell times, *h*^(*n*)^(*t*), is then calculated by $h^{\left(n\right)}(t)=dP_{6}(t)/dt$. From Equation (21), *h*^(*n*)^(*t*) has the form:

(22) $$h^{\left(n\right)}(t)=k_{10}P_{5}(t)$$

Assuming that the spontaneous opening and closing of mRNA base pairs next to the mRNA-entry channel of the ribosome follow rapid equilibrium kinetics, the overall distribution of dwell times, *h*(*t*), is an average over all possible mRNA-duplex conformational dependent distributions:

(23) $$h(t)=\sum_{n=0}^{\infty }\left[h^{\left(n\right)}(t)f_{\text{O}}^{\left(n\right)}\right]$$

Since $h^{\left(n\geq 3\right)}(t)=h^{\left(3\right)}(t)$ and $\sum_{n=0}^{\infty }f_{\text{O}}^{\left(n\right)}=1$, Equation (23) can be rewritten as:

(24) $$h(t)=\sum_{n=0}^{2}\left[h^{\left(n\right)}(t)f_{\text{O}}^{\left(n\right)}\right]+h^{\left(3\right)}(t)\left(1-\sum_{n=0}^{2}f_{\text{O}}^{\left(n\right)}\right)$$

Here, we solve Equations (11)–(21) numerically by using Runge-Kutta method to obtain *P*_5_(*t*). Then, using Equations (22) and (24) we calculate *h*(*t*).

### 4.5. Choice of Parameter Values

From Equations (7)–(10) it is seen that in order to calculate the probability of the effective translocation, $P_{\text{E}}^{\left(n\right)}$, during translation through the mRNA duplex, we require values of four fundamental parameters $E_{\text{PE}}^{\text{(}50S\text{)}}$, *Z*_max_, *b* and *E*_bp_, the choice of which is discussed as follows. As done before [13], we take $E_{\text{PE}}^{\left(50S\right)}$ = 9*k*_B_*T* (see also Supplementary materials) (With the value of $E_{\text{PE}}^{\left(50S\right)}$ = 9*k*_B_*T*, from Equation (2) we obtain that when translating through the single-stranded mRNA the probability of the effective translocation is calculated to be nearly 1, as expected). The maximum extension of the single-stranded mRNA containing one nucleotide under the pulling force *F* is taken to be *Z*_max_ = 0.58 nm, as done for the single-stranded DNA by Lionnet *et al.* [48]. For the single-stranded DNA, Smith *et al.* [49] reported a Kuhn length of 1.6 nm, Cui *et al.* [50] demonstrated that a Kuhn length of 0.59 nm fits the experimental data well, while Lionnet *et al.* [48] showed a Kuhn length of 2.46 nm. Thus, it is expected that the Kuhn length of the single-stranded mRNA should be in the range of *b* = 0.59–2.46 nm. For the calculation, we take *b* = 2 nm throughout. From the nearest-neighboring thermodynamic model for RNA duplex stability [51], *E*_bp_ is estimated to be about 3–3.5*k*_B_*T* for the mRNA duplex.

From Equations (11)–(24) we see that in order to calculate the distribution of dwell times, besides $P_{\text{E}}^{\left(n\right)}$ we also requires values of rate constants *k*_1_–*k*_11_ and *k_r_* defined in Figure 9. As this work focuses mainly on the theoretical studies of the distribution of dwell times and pausing which are compared with the *in vitro* single molecule experimental data [6], we take *in vitro* values of these rate constants in the calculations. From *in vitro* smFRET and biochemical data, the values of the rate constants are chosen as follows. The *in vitro* smFRET data showed that in the absence of EF-G, the rate of the spontaneous transition from State C0 to State H0 (see Figure 8) is about *k*_01_ = 0.27–3 s^–1^ [14,15,16,17,18]. On the other hand, when EF-G∙GDPNP binds to the ribosomal complex with deacylated tRNA^fMet^ bound to the 30S P site, the rate of the transition from classical non-rotated to hybrid state is increased by about 2.3-fold [18].Thus, it is estimated that *k*_1_ = 2.3*k*_01_ = 0.62–7 s^–1^. In the calculation, we take *k*_1_ = 1 s^–1^ (see Table 1). As EF-G∙GTP binding shifts the equilibrium of the pretranslocation ribosomal complex to and stabilizes the hybrid state [17,18,19], we take *k*_2_ = 0 (see Table 1). From the *in vitro* biochemical data for the rate of the ribosomal unlocking [20], we take *k*_3_ = 20 s^–1^ here (see Table 1). As the *in vitro* biochemical data showed that after the ribosomal unlocking the mRNA movement occurs rapidly [20], we take *k*_4_ having a large value, e.g., 100 s^–1^ (see Table 1) (Since the further increase of the value of *k*_4_ has nearly no effect on our results and the reverse intersubunit rotation facilitated by ribosomal unlocking occurs rapidly, for simplicity of treatment, we take the same large value of *k*_4_ = 100 s^–1^ for both the case of translocation without mRNA unwinding and that with mRNA unwinding). From other *in vitro* biochemical data [21,22,23,24,25,26], we have values of rate constants *k*_5_–*k*_11_ as shown in Table 1. It is important to note that with the above values of *k*_1_–*k*_11_, using Equations (S1) and (S2) the calculated rate of translation through the single-stranded mRNA at saturating concentration of the ternary complex is 0.67 s^–1^, which is consistent with the *in vitro* single molecule experimental data of Wen *et al.* [6,7]. The choice of the value of *k_r_* is discussed as follows. Recent *in vitro* single molecule experimental data showed that the lifetime of EF-G∙GDP bound to the pretranslocation state is about 20-fold longer than that bound to the post-translocation state [27]. On the other hand, from Table 1 it is seen that the rate of EF-G∙GDP release from the post-translocation state is *k*_5_ = 20 s^–1^. Thus, we take the rate of EF-G∙GDP release from the pre-translocation state to be $k_{r}=k_{5}/20$ = 1 s^–1^ (see Table 1).

Note that the experimental data in another work of Wen and colleagues (see Supplementary Figure 3 in Reference [7]) showed that in the range of 0.1–1 µM EF-G and in the range of 0.07–0.7 µM ternary complex, the rates of the ribosome translation through the mRNA duplex have a nearly constant value, implying that EF-G is saturating at concentration ≥0.1 µM and the ternary complex is saturating at concentration ≥0.07 µM. Thus, to compare our theoretical data with the experimental data presented in Wen *et al.* [6] where the concentration of EF-G was in the range of 0.1–1 µM and the concentration of the ternary complex was in the range of 0.07–0.7 µM, we consider that both EF-G and the ternary complex are at saturating concentrations.

## 5. Conclusions

In summary, the distributions of dwell times for the translation through the mRNA duplex are theoretically studied, providing quantitative explanations of the available single molecule optical trapping data. We show how the mRNA duplex can induce long translational pauses, how the presence of SD-like sequence can induce translational arrests, how the long paused or arrested ribosomes can be rescued, *etc.* Moreover, we compare the characteristics of these pauses or arrests induced by the mRNA duplex and/or SD-like sequence with those occurred at hungry codons and those induced by the interaction of the specific nascent chain sequence with the ribosome exit tunnel.

## Supplementary Materials

Supplementary materials can be found at http://www.mdpi.com/1422-0067/16/10/23723/s1.
