# Supplementary material for: Dwell-Time Distribution, Long Pausing and Arrest of Single-Ribosome Translation through the mRNA Duplex
Source: Int J Mol Sci. 2015 Oct 9;16(10):23723–44. doi: 10.3390/ijms161023723 (PMC4632723; doi:10.3390/ijms161023723)
Supplement: Supplementary file 1 [file ijms-16-23723-s001.pdf]

Supplementary Information

Determining the Value of $E_{\mathrm{PE}}^{(50S)}$

From Figure 2 for the translation through the single-stranded mRNA with $P_{\mathrm{E}} = 1$, the mean time of an elongation cycle under saturating concentrations of EF-G·GTP and the aminoacyl-tRNA·EF-Tu·GTP ternary complex is calculated by

$$\tau = \frac{k_1 + k_2 + k_3}{k_1 k_3} + \frac{1}{k_4} + \frac{1}{k_5} + \frac{k_7 + k_{-7} + k_8}{k_7 k_8} + \frac{1}{k_9} + \frac{1}{k_{10}} + \frac{1}{k_{11}} \tag{S1}$$

The translation rate is calculated by

$$v_0 = \frac{1}{\tau} \tag{S2}$$

Then, consider the translation through the mRNA duplex, with $P_{\mathrm{E}} < 1$. Based on Figure 2, for the mRNA duplex with n base pairs next to the mRNA-entry channel of the ribosome being open spontaneously the translation rate under saturating concentrations of EF-G·GTP and the ternary complex is calculated by

$$v^{(n)} = \left[\tau P_{\mathrm{E}}^{(n)} + \tau P_{\mathrm{E}}^{(n)}\left(1 - P_{E}^{(n)}\right) + \tau P_{\mathrm{E}}^{(n)}\left(1 - P_{\mathrm{E}}^{(n)}\right)^2 + \ldots + \tau_F\left(1 - P_{\mathrm{E}}^{(n)}\right) + \tau_F\left(1 - P_{\mathrm{E}}^{(n)}\right)^2 + \ldots\right]^{-1} \tag{S3}$$

where $P_{\mathrm{E}}^{(n)}$ is calculated by Equations (1)–(4), τ is calculated by Equation (S1) and τ_F is calculated by

$$\tau_F = \frac{k_1 + k_2 + k_3}{k_1 k_3} + \frac{1}{k_4} + \frac{1}{k_{\mathrm{r}}} \tag{S4}$$

Equation (S3) can be rewritten as

$$v^{(n)} = \frac{1}{\tau + \tau_F\left(1 - P_{\mathrm{E}}^{(n)}\right) / P_{\mathrm{E}}^{(n)}} \tag{S5}$$

The mean translation rate is then calculated by

$$v = \sum_{n=0}^{\infty}\left(v^{(n)} f_{\mathrm{O}}^{(n)}\right) \tag{S6}$$

Since $v^{(n\geq 3)} = v^{(3)}$ and $\sum_{n=0}^{\infty} f_{\mathrm{O}}^{(n)} = 1$, Equation (S6) can be rewritten as

$$v = \sum_{n=0}^{2}\left(v^{(n)} f_{\mathrm{O}}^{(n)}\right) + v^{(3)}\left(1 - \sum_{n=0}^{2} f_{\mathrm{O}}^{(n)}\right) \tag{S7}$$

It is seen that when $P_{E}^{(n)} = 1$, Equations (S5) and (S6) or (S7) are reduced to Equation (S2). It is also noted that if the time of slow EF-G·GDP release from the pre-translocation State F is close to the time of fast EF-G·GDP release from the post-translocation state plus the time from the binding of the ternary complex through the peptidyl transfer, *i.e.*, $\tau \approx \tau_F$, Equations (S5) and (S6) or (S7) are reduced to $v \approx \sum_{n=0}^{\infty}\left(v_0 P_{\mathrm{E}}^{(n)} f_{\mathrm{O}}^{(n)}\right)$, *i.e.*,

$$v(F) \approx v_0 \left\{ \sum_{n=0}^{2} \frac{1}{1+\exp\left[-\left(E_{\mathrm{PE}}^{(50S)}-(3-n)E_{\mathrm{bp}}\right)/k_{\mathrm{B}}T\right]} f_{\mathrm{O}}^{(n)} + \sum_{n=3}^{\infty} \frac{1}{1+\exp\left(-E_{\mathrm{PE}}^{(50S)}/k_{\mathrm{B}}T\right)} f_{\mathrm{O}}^{(n)} \right\} \qquad \text{(S8)}$$

Equation (S8) is the same as that used in the previous work [13]. It is noted here that with values of the rate constants given in Table 1, from Figure 2 it is calculated that the mean time of transition from State POST to State C at saturating ternary complex, $\tau_{POST\to C} = 1/k_5 + (k_7 + k_{-7} + k_8)/(k_7 k_8) + 1/k_9 + 1/k_{10} + 1/k_{11}$, is comparable to the time $\tau_r = 1/k_r$. Thus, as seen from Equations (S1) and (S4), the approximation $\tau \approx \tau_F$ is valid.

As done in the previous work [13], for fixed $Z_{\max}$ = 0.58 nm/base, E_{bp} = 3$k_{\mathrm{B}}T$ and b = 1.85 nm for mRNA duplex hpVal$_{\mathrm{GC50}}$, using Equation (S8) the experimental data on the rate of the ribosome translation through mRNA duplex hpVal$_{\mathrm{GC50}}$ *vs.* the pulling force F [7] can be fitted well, as shown in Figure S1, where the parameters for the ribosome are $E_{\mathrm{PE}}^{(50S)}$ = 9$k_{\mathrm{B}}T$ and v_0 = 0.42 codons/s. Similarly, for $Z_{\max}$ = 0.58 nm/base, E_{bp} = 3.3$k_{\mathrm{B}}T$ and b = 0.72 nm for mRNA duplex hpVal$_{\mathrm{GC100}}$, and $E_{\mathrm{PE}}^{(50S)}$ = 9$k_{\mathrm{B}}T$ and v_0 = 0.42 codons/s for the ribosome, using Equation (S8) the theoretical data on the rate of the ribosome translation through mRNA duplex hpVal$_{\mathrm{GC100}}$ are also in good agreement with the experimental data [7] (Figure S1). It is noted here that the base-pairing energies E_{bp} = 3$k_{\mathrm{B}}T$ for hpVal$_{\mathrm{GC50}}$ and E_{bp} = 3.3$k_{\mathrm{B}}T$ for hpVal$_{\mathrm{GC100}}$ are consistent with those estimated by using the nearest-neighboring thermodynamic model for the RNA duplex stability [51]. The maximum extension of the single-stranded mRNA containing onebaseunder the pulling force, $Z_{\max}$ = 0.58 nm/base, is the same as that used in the literature (see main text). The Kuhn length b = 1.85 nm for hpVal$_{\mathrm{GC50}}$ and b = 0.72 nm for hpVal$_{\mathrm{GC100}}$ are consistent with those (b = 0.6–2.5 nm) estimated in the literature (see main text). Moreover, since the Kuhn length is dependent on the persistence length that is sequence dependent [52], it is understandable that the two duplexeshpVal$_{\mathrm{GC50}}$and hpVal$_{\mathrm{GC100}}$ have different Kuhn lengths.

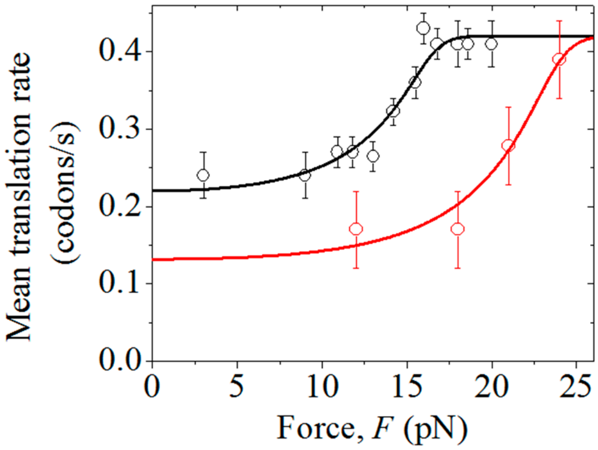

Figure S1. Mean rate of translation through mRNA duplexes *vs.* the pulling force F to unzip the duplexes. Solid lines are theoretical results and symbols are experimental data taken from Qu *et al.* [7] (reproduced with permission from Nature). The black line and symbols are for mRNA duplex hpVal$_{\mathrm{GC50}}$, while the red line and symbols are for mRNA duplex hpVal$_{\mathrm{GC100}}$.

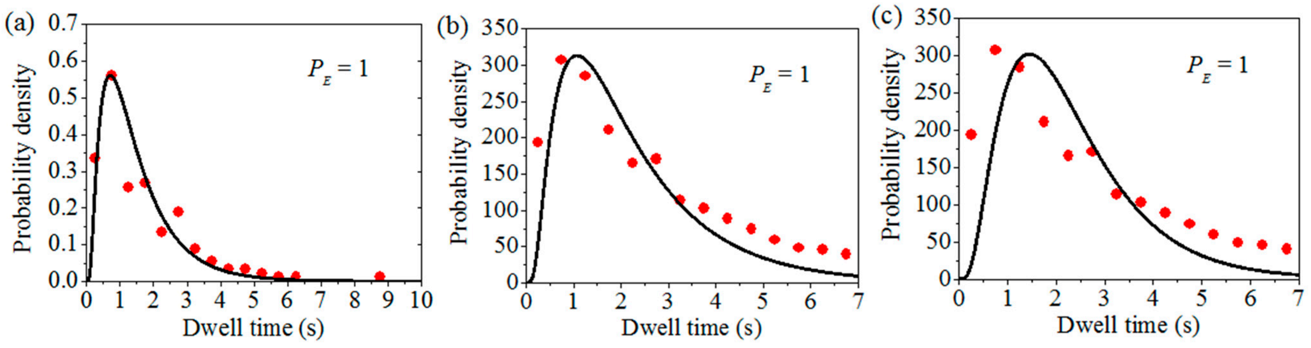

Figure S2. Distributions of dwell times calculated by taking $P_E = 1$ (black lines), which corresponds to the translation through the single-stranded mRNA. In order to make a direct comparison with the experimental data, we multiply the calculated distribution of dwell times, $h(t)$, by a constant C. Red dots are experimental data taken from Wen *et al.* [6]. (**a**) With values of rate constants k_1–k_{11} as given in Table 1. The experimental data are taken from Figure 4a in Wen *et al.* [6]; (**b**) With values of rate constants k_1–k_{11} given in Table 1 being divided by 1.5. The experimental data are taken from Figure S5a in Wen *et al.* [6]; (**c**) With rate constant of the translocation step, k_4, given in Table 1 being divided by 80 while with values of other rate constants as given in Table 1. The experimental data are taken from Figure S5a in Wen *et al.* [6] (reproduced with permission from Nature).

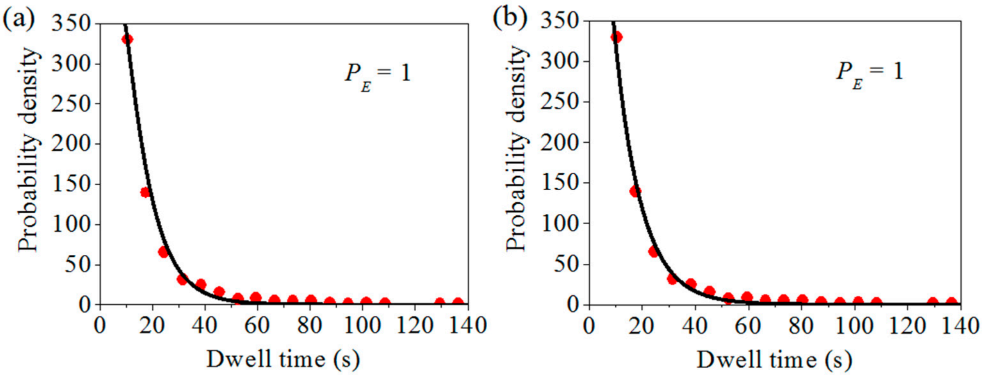

Figure S3. Distributions of dwell times with long durations (>7 s) calculated by taking $P_E = 1$ (black line), which corresponds to the translation through the single-stranded mRNA. In order to make a direct comparison with the experimental data, we multiply the calculated distribution of dwell times, $h(t)$, by a constant C. Red dots are experimental data taken from Figure S5b in Wen *et al.* [6] (reproduced with permission from Nature). (**a**) With values of rate constants k_1–k_{11} given in Table 1 being divided by 9; and (**b**) With rate constant of the translocation step, k_4, given in Table 1 being divided by 1000 while with values of other rate constants as given in Table 1.

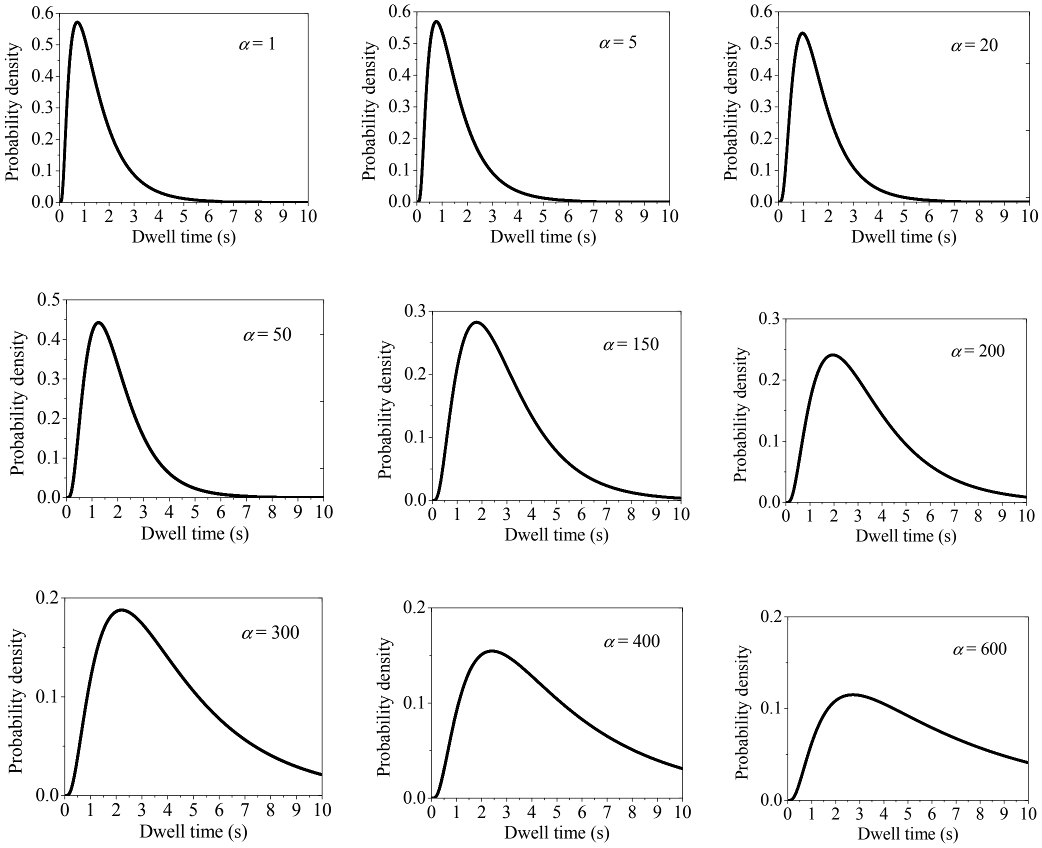

Figure S4. Normalized distributions of dwell times calculated by taking $P_E = 1$, which corresponds to the translation through the single-stranded mRNA, but with different values of α, where k_4 is replaced by k_4/α.

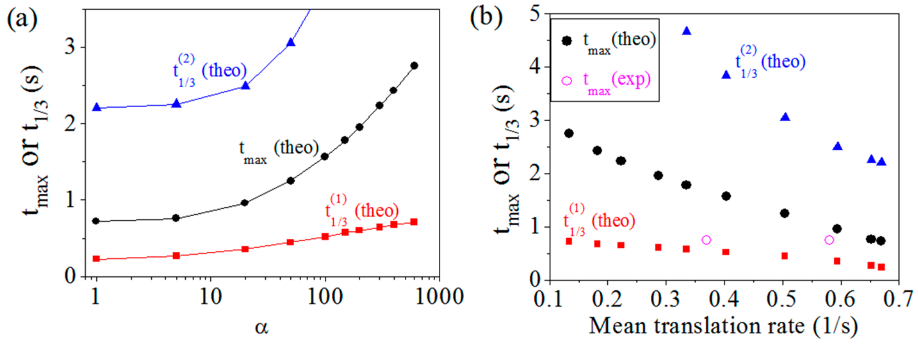

Figure S5. Dwell time, t_{max}, at which the maximum value of the distribution occurs, dwell time, $t_{1/3}^{(1)}$, at which the distribution increases from zero to the value equal to one third of the maximal value, and dwell time, $t_{1/3}^{(2)}$, at which the distribution decreases from the maximal value to the value equal to one third of the maximal value, *vs.* α (left panel, **a**), which are obtained from Figure S4. The right panel (**b**) corresponds to t_{max}, $t_{1/3}^{(1)}$ and $t_{1/3}^{(2)}$ *vs.* the translation rate v_0, where the theoretical data for v_0 are calculated by using Equations (S1) and (S2). The two experimental data for t_{max} are taken from and those for v are calculated from the two distributions of dwell times shown in Figure 4a and Figure S5a of Wen *et al.* [6].

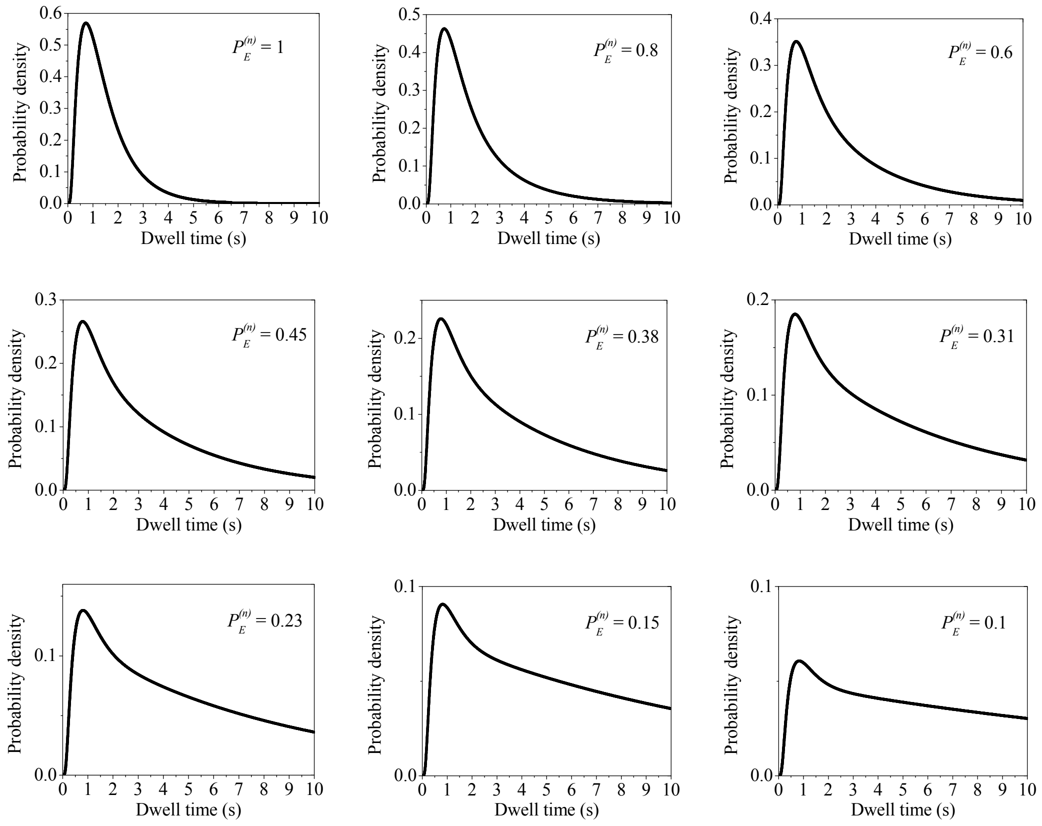

Figure S6. Normalized distributions of dwell times, $h^{(n)}(t)$ for different values of $P_{\rm E}^{(n)}$.

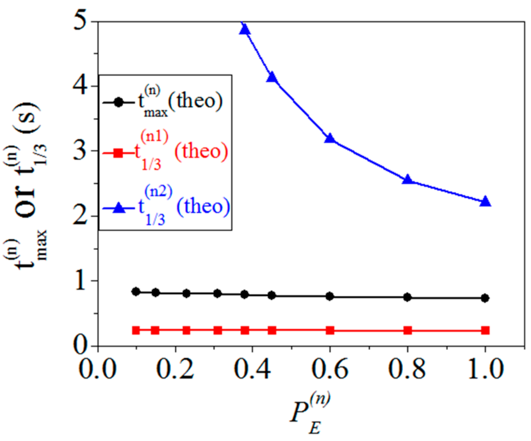

Figure S7. Dwell time, $t_{\rm max}^{(n)}$, at which the maximum value of the distribution $h^{(n)}(t)$ occurs, dwell time, $t_{1/3}^{(n1)}$, at which the distribution $h^{(n)}(t)$ increases from zero to the value equal to one third of the maximal value, and dwell time, $t_{1/3}^{(n2)}$, at which the distribution $h^{(n)}(t)$ decreases from the maximal value to the value equal to one third of the maximal value, *vs.* the effective translocation probability $P_{\rm E}^{(n)}$, which are obtained from Figure S6.
